# Compositional Signatures of Conventional, Free Range, and Organic Pork Meat Using Fingerprint Techniques

**DOI:** 10.3390/foods4030359

**Published:** 2015-08-25

**Authors:** Gislene B. Oliveira, Martin Alewijn, Rita Boerrigter-Eenling, Saskia M. van Ruth

**Affiliations:** 1 RIKILT, Wageningen University and Research Centre, P.O. Box 230, Wageningen 6700 AE, The Netherlands; E-Mails: gbremer@hotmail.com (G.B.O.); martin.alewijn@wur.nl (M.A.); rita.boerrigter-eenling@wur.nl (R.B.-E.); 2 Food Quality and Design Group, Wageningen University, P.O. Box 17, Wageningen 6700 AA, The Netherlands

**Keywords:** authenticity, ESI-MS/MS, fatty acid analysis, fraud, profiling, PTR-MS

## Abstract

Consumers’ interest in the way meat is produced is increasing in Europe. The resulting free range and organic meat products retail at a higher price, but are difficult to differentiate from their counterparts. To ascertain authenticity and prevent fraud, relevant markers need to be identified and new analytical methodology developed. The objective of this pilot study was to characterize pork belly meats of different animal welfare classes by their fatty acid (Fatty Acid Methyl Ester—FAME), non-volatile compound (electrospray ionization-tandem mass spectrometry—ESI-MS/MS), and volatile compound (proton-transfer-reaction mass spectrometry—PTR-MS) fingerprints. Well-defined pork belly meat samples (13 conventional, 15 free range, and 13 organic) originating from the Netherlands were subjected to analysis. Fingerprints appeared to be specific for the three categories, and resulted in 100%, 95.3%, and 95.3% correct identity predictions of training set samples for FAME, ESI-MS/MS, and PTR-MS respectively and slightly lower scores for the validation set. Organic meat was also well discriminated from the other two categories with 100% success rates for the training set for all three analytical approaches. Ten out of 25 FAs showed significant differences in abundance between organic meat and the other categories, free range meat differed significantly for 6 out of the 25 FAs. Overall, FAME fingerprinting presented highest discrimination power.

## 1. Introduction

Until recently, the consumer choice for food was based on sensory characteristics and aspects of lifestyle, diet, religious, and health issues [1,2]. However, nowadays, considerations about animal welfare and environmental consciousness also have influence on this choice, driven by perceived mistrust in food originating from the conventional production [3,4,5,6,7,8,9,10] or just to express a sense of self and improve psychological well-being [9].

In part, attention has been raised because of issues regarding the sustainability of the food production chain, and, consequently, of the planet, which are related to alarms about climate change, resource depletion, and economic recession [11]. The government’s concern is translated into political strategies which include animal protection and welfare topics, and have been published by the European Union for the period 2012–2015 [12].

In a few nations, initiatives have been developed to communicate with consumers which products have been produced in a more sustainable or animal–welfare considerate way than the mainstream products. In the Netherlands, in 2007, the Dutch Association for Animal Protection created a label called “Better Life” (http://beterleven.dierenbescherming.nl/), which distinguishes the system of meat production in three levels according to the animal welfare, ranging from relatively minor improvements over conventional production to the highest level, which involves also the organic specification. This kind of initiative is causing differentiation in the meat market but also creating opportunity for fraud, which is a serious issue for emerging markets.

It is noteworthy that consumers are not able to verify whether a product is organic or not even after consumption, which makes consumer confidence an important issue [13]. A recent study pointed to the label’s unreliability as one of the reasons why consumers with willingness to pay more for animal welfare friendly products do not do so [14]. “Food authentication” is the process of verifying if a food complies with its label’s description [15,16,17].

Effects of housing conditions (ambient temperature, floor type, space allowance, *etc.*), outdoor access and free range in pig rearing systems can influence physical activity and feed requirement affecting the animal performance, carcass, and meat quality [18,19]. The contrast can be even greater in the case of organic production, which has stricter rules compared to the conventional breeding methods, e.g., regarding the minimum space required for the animal, diet, and use of antibiotic growth promoters and genetically modified feed [18].

The influences mentioned above may provide analytical clues. For example, the fatty acids composition is largely dependent on the animal’s diet [1,20,21,22]. It makes this approach useful in many studies aiming for meat authentication [23,24,25,26], including differentiation between conventional and organic production [27,28,29,30]. As a drawback, this technique requires sample pretreatment, skilled personnel, and destruction of the sample [31].

Advances in mass spectrometry (MS) have also broadened its usability, being increasingly useful as an authentication method for different products [32,33] from where some minor compositional changes can be easily assessed. Direct infusion mass spectrometry (DI-MS) is a technique based on the injection of a sample in the ion source without or with small pretreatment. It reduces the analysis time, being especially useful in case of high throughput screenings [34]. DI-MS can be coupled with electrospray ionization (ESI), a typical soft ionization that preserves the molecule integrity and which is suitable for large molecules e.g. proteins, peptides, polysaccharides and triglycerides [35,36]. ESI makes the liquid in which the analyte is contained disperse by electrospray to a fine aerosol. The droplets shrink as the solvent evaporates till solvated ions desorb from their surface [34]. Recently, MS techniques has been shown useful to detect improper blends of meats from different species in processed food matrices or to identify animal species in different kinds of processed meat [37,38].

Meat includes also a complex mixtures of volatile constituents [39] which has also been used as a means of food authentication [10,40]. A feasible way to analyze the volatile profile of a meat sample is by means of gas chromatography and electronic nose [41]. However, Proton Transfer Reaction Mass Spectrometry (PTR-MS) [34,42], a technique which also allows a rapid analysis of volatiles, has been successfully applied in food analysis aiming at authentication in general [43,44,45,46,47,48,49,50]. Through this technique, the organic volatile compounds with proton affinity higher than water contact and react with an intense and pure H3O+ ion beam generated in pure water vapor, resulting in ions often with no or minor fragmentation, which are detected by a quadrupole mass analyzer creating a fingerprint of the sample investigated [42,51]. High sensitivity (in order of parts per trillion by volume or pptv), instantaneously results, no need for sample pre-treatment and sample preservation are the main advantages of PTR-MS, while the impossibility to precisely identify the compound through its m/z is the main disadvantage of PTR-MS [34]. Although PTR-MS has being used for authentication purposes (origin) in many food matrices, in the case of meat it has been applied only in view of quality control during storage and management of spoilage processes in meat samples [52,53].

Fatty acid profiling, non-volatile compound profiling by ESI-MS/MS, and volatile compound profiling by PTR-MS analysis can provide a comprehensive and multivariate description of food samples, analogous to a fingerprint [31,54], and they are, therefore, useful for authentication purposes.

The objective of the current pilot study was to characterize commercial pork meats of different animal welfare classes by their fatty acid, non-volatile compounds (ESI-MS) and volatile compounds (PTR-MS) fingerprints in order to elucidate analytical markers useful for authentication methods. The wealth of data generated by the techniques was processed by chemometric tools in order to compare the analytical fingerprints of the meat of the different categories.

## 2. Experimental Section

### 2.1. Samples

A total of 41 commercial samples of belly pork meat (13 conventional, 15 from the category one star or “free range” of the animal welfare program and 13 from organic production) were supplied by a large meat producing company in the Netherlands. The sample set was balanced for males/females and all samples originated from different males and primipara females bred in the Netherlands and slaughtered in April 2011. The productions were in accordance with the requirements set by the animal welfare program “Beter Leven” in the Netherlands. Pork belly samples were cut by professional staff of the meat company according to regular in-company procedures. Samples were transported to Wageningen University and Research Centre in the Netherlands.

### 2.2. Chemical and Reagents

Chloroform, dichloromethane, and isooctane were supplied by Biosolve (Dieuze, France). Potassium hydroxide, formic acid and water free Na_2_SO_4_ was obtained from Merck (Darmstad, Germany). Water LC/MS grade was purchased from Thermo Scientific (Rockford, IL, USA) and MeOH from Actu-all (Oss, The Netherlands).

### 2.3. Fat Extraction

The fat part of the meat was ground using use of liquid nitrogen and a Grindomix (GM 200, Retsch, Düsseldorf, DE, Germany). Approximately 50 mL chloroform was added to 10 g of fat and an excess of water free Na_2_SO_4_. The mixture was blended for 1 min and the suspension was filtrated (Whatmann^®^ 597, 240 mm diameter, GE Healthcare, Maidstone, Kent, UK). The clear filtrated solution was evaporated at 50 °C under a N2 stream and the fat was stored in a freezer at −20 °C.

### 2.4. Fatty Acid Methyl Ester (FAME) Analyses

Approximately 100 mg of the pure fat was used for the fatty acid analyses. Primarily the fat was melted at 45 °C and centrifuged for 20 sec to remove excess water before weighing and homogenized with an Ultra-Turrax^®^ T25 (Ika, Staufen, DE, Germany). For the methylation 60 mg of fat was dissolved in 5 mL of isooctane. 200 µL of KOH 24% in MeOH was added and the mixture was shaken for one minute. The isooctane layer was transferred to a vial and the FAMEs were determined by gas chromatography in a Focus GC model gas chromatograph (Thermo Fisher Scientific Inc., Waltham, MA, USA) fitted with a flame-ionization detector and split-splitless injector port set at 270 and 250 °C, respectively. The split ratio was 1:30. Chromatographic separation of FAMEs was performed on a CP-Select CB for FAME capillary column (50 m × 0.25 mm inner diameter; Varian, Palo Alto, CA, USA). Helium (1 mL/min) was used as a carrier gas, and the oven was programmed as follows: initial temperature 100 °C increased at 5 °C/min to 230 °C and held for 9 min. The sample volume injected was 1 µL. Fatty acids were identified by their retention times according to those found in the FAME standard (Supelco 37, Sigma-Aldrich, St. Louis, MO, USA). All samples were analyzed in duplicate, and the results were expressed as normalized peak areas (%). Data used were the average value of the two replicates of each sample.

### 2.5. ESI-MS/MS

The fat was melted at 45 °C and centrifuged for 20 sec to remove excess water. Approximately 50 mg of fat was weighed and solved in 10 mL dichloromethane and the solution was diluted 20 times in the mobile phase MeOH/DCM (80:20). The samples were analyzed on an Acella LC system (Thermo Scientific, Bremen, Germany) coupled to an Exactive (Orbitrap-) Mass Spectrometer (MS) system (Thermo Scientific). Instead of a LC column, a ~1 m length of PTFE tubing (150 µm i.d.) was used to make a connection between the auto sampler and the MS system. 5 µL of sample was injected and a flow of 300 µL/min of LC/MS grade water containing 0.1% formic acid was used to transport the sample from the auto sampler loop to the MS system. The MS system was fitted with a HESI-II electrospray source. The samples were measured in positive mode, using a resolving power of 100.000 (at m/z 200) at a scan rate of ~1.2 Hz. Data was collected in the range m/z 120–1200. The run time of each sample was 2 min. To minimize sample carry-over, a blank solvent was injected between each replicate.

### 2.6. PTR-MS

Five grams of ground meat (without fat) was placed in a 250 mL glass bottle (Schott North America Inc., Elmsford, NY, USA) which were equilibrated by being immersed in a water bath for 20 min at 45 °C. Two replicates for each sample were analyzed, randomizing the order analysis and the result was based on the average of the two replicates. The headspace was drawn at a rate of 60 mL/min, 32 mL/min of which was led into the PTR-MS (Ionicon, Innsbruck, Austria). A constant drift voltage of 600 V was maintained in the reaction chamber. MS data were collected over the mass range m/z 20-160 using a dwell time of 200 ms. The average of the 3rd-5th cycle for each replicate sample was used for data analysis after preliminary studies indicating stabilization of the signal in these cycles. Blank samples were measured between all samples for background corrections. Headspace concentrations were calculated considering background and transmission corrections.

### 2.7. Statistical Analysis

Three datasets (from each instrumental technique) were organized in 41 rows (number of samples analyzed) and as many columns as necessary for the number of informative variables. The data pre-treatment included normalization of the replicate averages by dividing by the sum of all variables for each sample. All models were created using Pirouette 4.5 (Bothell, WA, USA) with 95% confidence level.

As a first step, Principal Component Analysis (PCA) was applied for each dataset as an unsupervised method to screen the multivariate data for outliers and to explore the presence of natural clustering [47]. PCA has as a goal to reconstruct the dataset and create new uncorrelated variables. The principal components are determined on the basis of the maximum variance criterion, with each one describing a maximum variance that is not modelled by the former components. Most of the data’s variance is contained in the first principal component and in the second component there is more information than in the third until as many principal components as needed are computed to explain an adequate portion of the variance. Usually, the majority of the variance can be described by means of two or three principal components, enabling visualization of the data by plotting the principal components against each other [48].

As a second step, Soft Independent Modeling of Class Analogy (SIMCA) was used as a supervised classification technique to develop classification models for each instrumental technique (a) using the three categories of pork belly samples (conventional, free range, and organic); and (b) comparison between organic and non-organic (conventional and free range) categories. SIMCA develops PCA models for each category (combined or not) aiming the classification of new (and unknown) samples. Several data pre-treatments were evaluated (none, log transformation, auto scale, mean center, and Pareto scale) to select the model with the highest correct prediction rates for classifying samples in the category of interest. To evaluate the models performance, the stratified dataset was randomly split into training (80%) and validation (20%) sets. The model to evaluate was recreated with only the samples from the training set, and the correct prediction rate of the samples in the validation set was used to evaluate the performance of the model.

Significant differences in fatty acids concentration were evaluated by ANOVA single factor using Excel 2010 (Microsoft Corp., Redmond, WA, USA) with 95% confidence level.

### 2.8. Variable Selection

Variables containing information for the classification were retained, whereas those encoding known noises were eliminated. In the case of FAME analysis, a group of the 25 most important according to high correlation coefficient (or modelling power), were used. For PTR-MS, the analysis excluded m/z 32 (O2) and m/z 37 (water cluster) and 11 other organic volatile compounds (m/z 33, 47, 49, 57, 63, 75, 113, 127, 129, 131 and 143), which are correlated with meat deterioration according to Mayr *et al.* [53].

## 3. Results and Discussion

### 3.1. Three Animal Welfare Categories

**Figure 1:**
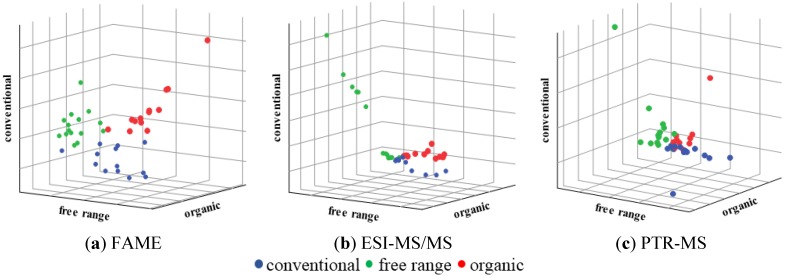
Graphical representation of sample distance to each class of the Soft Independent Modeling of Class Analogy (SIMCA) models for samples of pork belly meat from the categories conventional, free range, and organic.

41 samples of pork belly meat were analyzed by FAME, ESI-MS/MS, and PTR-MS profiles and subjected to a PCA analysis. The visual evaluation of the PCA showed that three outliers needed to be removed from both ESI-MS/MS and PTR-MS datasets. The greatest tendency of sample natural clustering within classes after PCA resulted from the FAME data (not shown). After this initial data evaluation SIMCA was applied and the same tendency was observed: FAME data using auto-scaling as a pre-processing method showed most distinct clustering (Figure 1a) followed by the ESI-MS/MS using auto-scaling for pre-processing (Figure 1b) and PTR-MS data using Pareto-scaling for pre-processing (Figure 1c).

The performance of the SIMCA models resulted in high percentages (from 93 to 100) of correct classification for the training set (Table 1). Table 1 also shows the evaluation of the model performance, where eight of nine samples were correctly classified for the FAME method as well as seven of nine samples were correctly classified for ESI-MS/MS method and eight of eight for the PTR-MS method.

The classification results in the training and validation sets show that the analysis of the FAME profile was most successful of animal welfare in the pork rearing system regarding to authentication issues, although ESI-MS/MS and PTR-MS also presented good results.

The percentages of individual fatty acids as well as their sums in different categories for conventional, free range and organic meat are shown in Table 2. The organic meat differed significantly (*p <* 0.05) from the other two categories for 10 out of the 25 fatty acids; free range meat did so for 6 out of the 25 fatty acids, and conventional meat differed only for two fatty acids significantly in abundance from the free range and organic meat samples. All three categories were differentiated significantly for their abundance in linoleic acid, alpha-linolenic acid, and eicosadienoic acid. The differences in fatty acid composition are the underlying cause for the successful prediction of the meat welfare categories using the FAME fingerprints. The group of monounsaturated fatty acids (MUFAs) were the most abundant (43%, 45% and 41% for conventional, free range and organic categories), followed by group the saturated fatty acids (Table 2). ANOVA indicates that the three categories of meat contain significantly (*p <* 0.05) different levels of polyunsaturated fatty acids (PUFAs), including the omega-3 series (Table 2).

The current results of the FAME profiling can be compared with results of Husak [30], who reported a study including FAME profiling of conventional, free range, and organic broilers. According to these authors, the FAME profiling showed the most significant difference in between broilers and organic broilers presented higher percentages of PUFAs. The differences observed could be, according to them, owing to the outdoor access (including grass and other organic materials) and the fact that free range animals also have outdoor access creates difficulty in positioning this category.

No other reports on differentiation of these three welfare categories by the techniques currently used or otherwise are available according to the authors’ knowledge.

**Table 1:** Percentage of samples of pork belly meat from the categories conventional, free range and organic correctly classified in the training set of the Soft Independent Modeling of Class Analogy (SIMCA) models.

	Fatty Acid Methyl Ester (FAME)						Electrospray ionization-tandem mass spectrometry (ESI-MS/MS)					Proton-transfer-reaction mass spectrometry (PTR-MS)				
	samples classified as						samples classified as					samples classified as				
All data					correct					correct					correct
	Conventional	Free range	Organic	Not classified	classification (%)	Conventional	Free range	Organic	Not classified	classification (%)	Conventional	Free range	Organic	Not classified	Classification (%)
Conventional	13	0	0	0	100	12	0	0	0	100	13	0	0	0	100
Free range	0	15	0	0	100	1	13	0	0	93	1	14	0	0	93
Organic	0	0	13	0	100	0	0	12	0	100	0	0	10	0	100
Average of training set					100					95.3					95.3
Model performance evaluation															
Training set					correct					correct					correct
	Conventional	Free range	Organic	Not classified	classification (%)	Conventional	Free range	Organic	Not classified	classification (%)	Conventional	Free range	Organic	Not classified	classification (%)
Conventional	10	0	0	0	100	9	0	0	0	100	10	0	0	0	100
Free range	0	12	0	0	100	0	11	0	0	100	1	11	0	0	92
Organic	0	0	10	0	100	0	0	9	0	100	0	0	8	0	100
Validation set															
Conventional	3	0	0	0	100	3	0	0	0	100	3	0	0	0	100
Free range	0	3	0	0	100	1	2	0	0	67	0	3	0	0	100
Organic	0	0	2	1	67	1	0	2	0	67	0	0	2	0	100
Average of validation set					89					78					100

**Table 2:** Fatty acid composition of pork belly meat from the categories conventional, free range, and organic expressed as percentage of normalized peak area.

Fatty acid	Conventional	Free Range	Organic
Myristoleic acid	0.02 ^a^ ± 0.01	0.02 ^a^ ± 0.00	0.01 ^b^ ± 0.00
Pentadecylic acid	0.06 ^a,b^ ± 0.02	0.06 ^a^ ± 0.01	0.07 ^b^ ± 0.02
Palmitic acid	25.32 ^a^ ± 1.10	25.37 ^a^ ± 0.91	22.98 ^b^ ± 1.86
Palmitoleic acid	2.09 ^a^ ± 0.40	2.10 ^a^ ± 0.29	1.59 ^b^ ± 0.21
Palmitoleic acid	0.01 ^a^ ± 0.00	0.01 ^a^ ± 0.01	0.01 ^a^ ± 0.00
Margaric acid	0.32 ^a^ ± 0.07	0.30 ^a^ ± 0.09	0.40 ^b^ ± 0.11
Heptadecanoic acid	0.23 ^a^ ± 0.05	0.23 ^a^ ± 0.08	0.27^a^ ± 0.08
Stearic acid	13.86 ^a,b^ ± 2.38	14.23 ^a^ ± 1.17	12.78 ^b^ ± 1.44
Trans elaidic acid	0.23 ^a^ ± 0.05	0.22 ^a,b^ ± 0.06	0.18 ^b^ ± 0.05
Oleic acid	36.79 ^a^ ± 3.04	38.93 ^b^ ± 2.05	35.97 ^a^ ± 1.56
Vaccenic acid	2.55 ^a^ ± 0.28	2.63 ^a^ ± 0.32	2.28 ^b^ ± 0.17
Unknown 20.4 min	0.04 ^a,b^ ± 0.01	0.04 ^a^ ± 0.01	0.03 ^b^ ± 0.01
Linoleic acid	14.97 ^a^ ± 3.64	12.58 ^b^ ± 2.17	18.98 ^c^ ± 2.98
Unknown 21.2	0.03 ^a^ ± 0.01	0.04 ^a^ ± 0.01	0.04 ^a^ ± 0.01
Gamma-linolenic acid	0.01 ^a,b^ ± 0.00	0.01 ^a^ ± 0.00	0.01 ^b^ ± 0.00
Alpha-linolenic acid	1.44 ^a^ ± 0.40	1.15 ^b^ ± 0.25	2.02 ^c^ ± 0.34
Eicosenoic acid	0.71 ^a^ ± 0.11	0.83 ^b^ ± 0.11	0.73 ^a^ ± 0.08
Eicosadienoic acid	0.52 ^a^ ± 0.08	0.51 ^b^ ± 0.07	0.70 ^c^ ± 0.10
Unknown 23.3 min	0.03 ^a,b^ ± 0.01	0.03 ^a^ ± 0.01	0.02 ^b^ ± 0.01
Unknown 23.5 min	0.03 ^a^ ± 0.01	0.03 ^a^ ± 0.01	0.03 ^a^ ± 0.01
Dihomo-gamma-linolenic acid	0.09 ^a^ ± 0.02	0.09 ^a^ ± 0.01	0.11 ^a^ ± 0.02
Eicosatrienoic acid + arachidonic acid	0.41 ^a^ ± 0.08	0.37 ^a^ ± 0.06	0.5 ^b^ ± 0.08
Adrenic acid	0.10 ^a^ ± 0.02	0.10 ^a^ ± 0.02	0.11 ^a^ ± 0.03
Docosapentaenoic acid (osbond acid)	0.02 ^a^ ± 0.01	0.01 ^b^ ± 0.00	0.02 ^a^ ± 0.00
Docosapentaenoic acid (clupanodonic acid)	0.12 ^a^ ± 0.03	0.12 ^a^ ± 0.05	0.15 ^b^ ± 0.03
∑ Saturated	39.56 ^a^ ± 2.63	39.95 ^a^ ± 1.48	36.24 ^b^ ± 2.35
∑ Monounsaturated	42.63 ^a^ ± 3.10	44.97 ^b^ ± 2.10	41.03 ^a^ ± 1.58
∑ Polyunsaturated	17.68 ^a^ ± 3.67	14.95 ^b^ ± 2.19	22.60 ^c^ ± 2.98
∑ Unknown	0.13 ^a^ ± 0.02	0.14 ^a^ ± 0.02	0.13 ^a^ ± 0.01
∑ Omega-3	1.97 ^a^ ± 0.41	1.64 ^b^ ± 0.26	2.67 ^c^ ± 0.35

ValVue*Values followed by the same letters in the same row do not differ significantly (*p* value > 0.05); Values shown for individual fatty acids correspond to the average of 13 samples from the conventional category, 15 samples from the free range category and 13 samples from the organic category; The FAs eicosatrienoic acid and arachidonic acid are reported together because they co-eluted in the chromatographic procedure.

**Figure 2:**
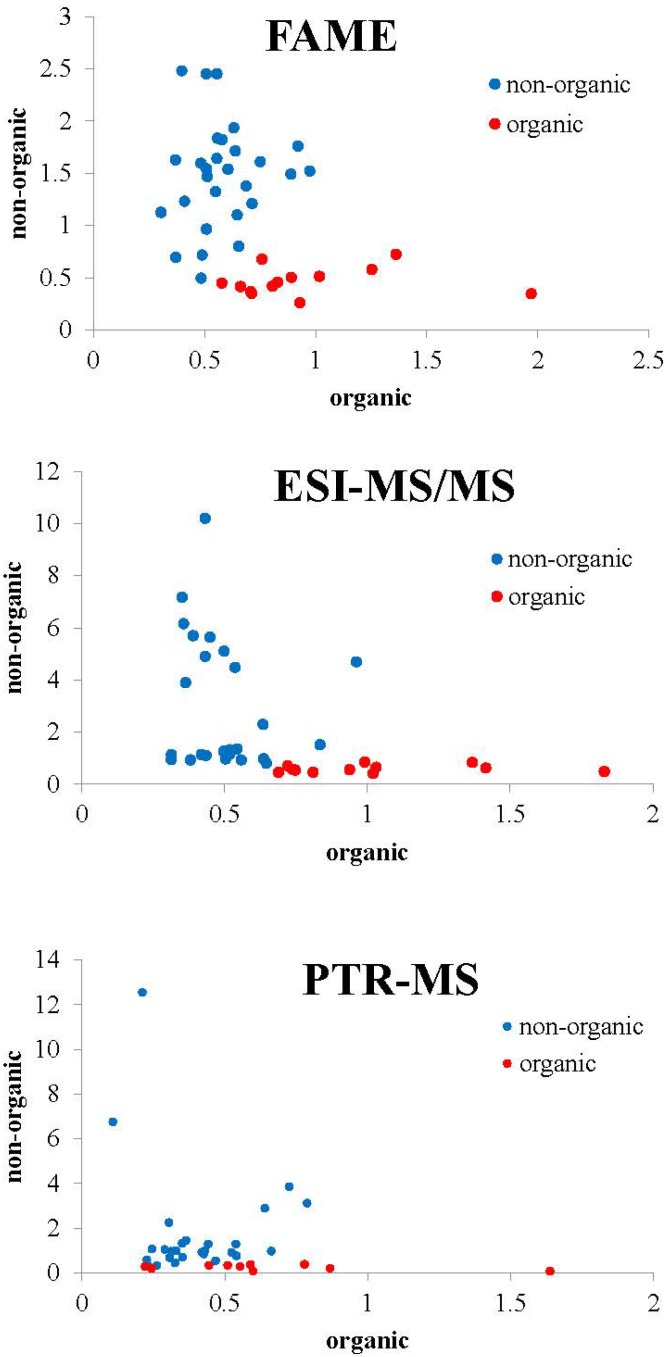
Graphical representation of sample distance to each class of the SIMCA models for samples of pork belly meat from the categories non-organic (conventional + free range) and organic.

**Table 3:** Percentage of samples of pork belly meat from the categories non-organic (conventional + free range) and organic correctly classified in the training set of the SIMCA models.

	Fatty Acid Methyl Ester (FAME)				Electrospray ionization-tandem mass spectrometry (ESI-MS/MS)				Proton-transfer-reaction mass spectrometry (PTR-MS)			
	samples classified as				samples classified as				samples classified as			
All data				correct				correct				correct
	Non-organic	Organic	Not classified	Classification (%)	Non-organic	Organic	Not classified	Classification (%)	Non-organic	Organic	Not classified	Classification (%)
Non-organic^a^	28	0	0	100	26	0	0	100	28	0	0	100
Organic	0	13	0	100	0	12	0	100	0	10	0	100
Average of training set				100				100				100
Model performance evaluation				correct				correct				correct
Training set	Non-organic	Organic	Not classified	classification (%)	Non-organic	Organic	Not classified	classification (%)	Non-organic	Organic	Not classified	classification (%)
Non-organic^a^	22	0	0	100	20	0	0	100	22	0	0	100
Organic	0	10	0	100	0	9	0	100	0	8	0	100
Validation set												
Non-organic^a^	6	0	0	100	6	0	0	100	6	0	0	100
Organic	0	3	0	100	1	2	0	67	0	1	1	50
Average of validation set				100				83				75

^a^ conventional and free range.

### 3.2. Organic Versus Conventional Meat

Since authentication of organic *versus* non-organic meat is also an important issue, new models were developed to differentiate organic meat from the other two categories. These two-class models are graphically presented in Figure 2.

The performance of these two-class SIMCA models (Table 3) resulted in excellent percentage (100%) of correct classification in the training set. Table 1 also shows the evaluation of the model performance, where nine of nine samples were correctly classified for the FAME method as well as eight of nine samples were correctly classified for ESI-MS/MS method and seven of eight for the PTR-MS method.

The two-class models highlight FAME as the most discriminating method to distinguish the organic production from other categories.

The percentages of individual fatty acids as well as their sums in different categories for non-organic and organic meat are shown in Table 4. Individually, ANOVA showed that 18 of 25 analyzed fatty acids presented significant differences between the categories organic and non-organic. The group of MUFAs was the most abundant (43% for the non-organic category and 40% for the organic category), followed by the saturated fatty acids. The levels of MUFAs and PUFAs, including the omega-3 series, were also higher and significantly different (*p <* 0.05) for organic meat when compared with non-organic meat in the case of the two-class models.

Many studies have been carried out on the authentication of organic meat and many authors have reached to similar conclusions in terms of FAME profile. Higher levels of PUFAs, including the omega-3 series seems to occur in organic lamb [29], pork [27], and broiler [28,30] meat, corroborating with our study and with a study of Lebret [18], who confirms that the meat quality can be affected by the feeding and rearing system. No studies have been reported on the application of ESI-MS/MS and PTR-MS for meat authentication according to animal welfare classes.

**Table 4:** Fatty acid composition of pork belly meat from the categories non-organic (conventional + free range) and organic expressed as percentage of normalized peak area.

Fatty acid	Non-Organic	Organic
Myristoleic acid	0.02 ^a^ ± 0.01	0.01 ^b^ ± 0.00
Pentadecylic acid	0.06 ^a^ ± 0.02	0.07 ^b^ ± 0.02
Palmitic acid	25.34 ^a^ ± 0.98	22.98 ^b^ ± 1.86
Palmitoleic acid	2.09 ^a^ ± 0.34	1.59 ^b^ ± 0.21
Palmitoleic acid	0.01 ^a^ ± 0.00	0.01 ^a^ ± 0.01
Margaric acid	0.31 ^a^ ± 0.08	0.40 ^b^ ± 0.11
Heptadecanoic acid	0.23 ^a^ ± 0.07	0.27 ^a^ ± 0.08
Stearic acid	14.06 ^a^ ± 1.81	12.78 ^b^ ± 1.44
Trans elaidic acid	0.23 ^a^ ± 0.06	0.18 ^b^ ± 0.05
Oleic acid	37.94 ^a^ ± 2.73	35.97 ^b^ ± 1.56
Vaccenic acid	2.59 ^a^ ± 0.30	2.28 ^b^ ± 0.17
Unknown 20.4	0.04 ^a^ ± 0.01	0.03 ^a^ ± 0.01
Linoleic acid	13.69 ^a^ ± 3.13	18.98 ^b^ ± 2.98
Unknown 21.2	0.03 ^a^ ± 0.01	0.04 ^a^ ± 0.01
Gamma-linolenic acid	0.01 ^a^ ± 0.00	0.01 ^b^ ± 0.00
Alpha-linolenic acid	1.29 ^a^ ± 0.35	2.02 ^b^ ± 0.34
Eicosenoic acid	0.77 ^a^ ± 0.12	0.73 ^a^ ± 0.08
Eicosadienoic acid	0.51 ^a^ ± 0.07	0.7 ^b^ ± 0.10
Unknown 23.3	0.03 ^a^ ± 0.01	0.02 ^b^ ± 0.01
Unknown 23.5	0.03 ^a^ ± 0.01	0.03 ^a^ ± 0.01
Dihomo-gamma-linolenic acid	0.09 ^a^ ± 0.02	0.11 ^b^ ± 0.02
Eicosatrienoic acid + arachidonic acid	0.39 ^a^ ± 0.07	0.5 ^b^ ± 0.08
Adrenic acid	0.1 ^a^ ± 0.02	0.11 ^a^ ± 0.03
Docosapentaenoic acid (osbond acid)	0.01 ^a^ ± 0.01	0.02 ^b^ ± 0.00
Docosapentaenoic acid (clupanodonic acid)	0.12 ^a^ ± 0.04	0.15 ^b^ ± 0.03
∑ Saturated	39.77^a^ ± 2.06	36.24 ^a^ ± 2.35
∑ Monounsaturated	43.88 ^a^ ± 2.77	41.03 ^b^ ± 1.58
∑ Polyunsaturated	16.22 ^a^ ± 3.15	22.60 ^b^ ± 3.00
∑ Unknown	0.14 ^a^ ± 0.02	0.13 ^a^ ± 0.01
∑ Omega-3	1.79 ^a^ ± 0.36	2.67 ^b^ ± 0.35

*Values followed by the same letters in the same row do not differ significantly (*p* value > 0.05); Values shown for individual fatty acids correspond to the average of 28 samples from the non-organic and 13 samples from the organic category; The FAs eicosatrienoic acid and arachidonic acid are reported together because they coeluted in the chromatographic procedure.

## 4. Conclusions

The results show that the fatty acids (FAME), non-volatile compounds (ESI-MS/MS), and volatile compounds (PTR-MS) profiles hold relevant information to distinguish pork meat of different animal welfare categories. Especially organic meat can be discriminated from the two other categories by a variety of variables. Relatively small changes in the breeding system in relation to animal welfare, as is the case of meat from free range conditions, remains a greater analytical challenge, which could be probably overcome with a higher number of samples from different (Dutch) producers and production periods.
